# Two new species of *Polyrhachis* from the subgenera *Myrma* and *Myrmhopla* (Hymenoptera: Formicidae) from China

**DOI:** 10.1093/jisesa/ieag070

**Published:** 2026-08-10

**Authors:** Chao Chen, Jialin Yu, Qiuhan Yang

**Affiliations:** Kunming Natural History Museum of Zoology, Kunming Institute of Zoology, Chinese Academy of Sciences, Kunming, Yunnan Province, China; Forestry College, Southwest Forestry University, Kunming, Yunnan Province, China; Business College, Shanghai Dianji University, Shanghai, China; Forestry College, Southwest Forestry University, Kunming, Yunnan Province, China

**Keywords:** biodiversity, oriental, morphology, foraging, sympatry

## Abstract

Based on the morphological characters of the worker caste, a taxonomic study of the ant genus *Polyrhachis* Smith 1857 from China was conducted. Two new species, *Polyrhachis* (*Myrma*) *aureodorsalis*, **sp. nov.** and *Polyrhachis* (*Myrmhopla*) *mangensis*, **sp. nov.** are described from Fujian and Yunnan Provinces, respectively. Detailed descriptions and high-resolution images for the new species are provided. Identification keys to the known Chinese species of the subgenera *Myrma* Billberg 1820 and *Myrmhopla* Forel 1915 are presented based on the worker caste. The ecological significance and potential morphological adaptations of the new species are briefly discussed.

## Introduction

The spiny ant genus *Polyrhachis*  Smith 1857 (Hymenoptera: Formicidae) is one of the largest ant genera in the world in number of species (Jaitrong et al. 2023). The taxonomic history of the genus *Polyrhachis* began with an unavailable use of the name by Swainson and Shuckard (1840). The genus was formally established later by Smith (1857), who designated *Formica bihamata* Drury 1773 as its type species. Wheeler (1911) subsequently treated *Polyrhachis* as a subgenus of *Myrma* for *Polyrhachis bihamata* and its allied species. It was Emery (1925) who later provided a foundational diagnosis for this subgenus and divided it into the *bihamata* and *lamellidens* groups based on mesosomal morphology. It is mainly distributed in tropical and subtropical areas of the Old World, New Guinea, and Australia (Kohout 2013, Mezger and Moreau 2016); spiny ants also vary wildly in their geographic range even within the same subgenus, with some species restricted to 1 locality while others are distributed across much of the Oriental and Australasian regions (Janicki et al. 2016, Guénard et al. 2017). Currently, 709 extant species, 82 subspecies, and 1 fossil species are known (Bolton 2025).

The genus *Polyrhachis* exhibits remarkable ecological diversity, particularly in its nesting strategies. Nesting habits are broadly categorized into 4 types: arboreal, lignicolous, terrestrial, and subterranean (Hung 1967, Bolton 1973). While nesting behavior is generally conserved at the subgenus level, a review by Robson and Kohout (2007) revealed that some species demonstrate notable plasticity, utilizing both subterranean and arboreal sites. A key trait associated with arboreal nesting is the incorporation of larval silk into carton nests, although the presence of spider silk in the nests of some species indicates that the origin of silk must be interpreted with caution (Robson and Kohout 2007). The genus encompasses variations in several key ecological strategies: recruitment can range from single to mass; nesting structures vary from monodomous to polydomous; colonies can exhibit either monogyny or polygyny; and mature colony sizes can range from small to very large (Dorow 1995).

The subgeneric classification of *Polyrhachis* has been refined over time. Dorow (1995) retained 12 subgenera, and Kohout (2010) added a further subgenus. The subgenus *Myrma* Billberg is notably widespread and species-rich, distributed across the Indo-Malayan, Oriental, Australasian, and Ethiopian regions (Kohout 2013). While its diversity has been partitioned into several species-groups (eg Emery 1925). Ecologically, species of *Myrma* are predominantly arboreal or lignicolous, nesting in plant cavities or constructing carton nests with larval silk, and they are often found foraging on tree trunks and foliage (Robson and Kohout 2007).

The subgenus *Myrmhopla* was established by Forel (1915), designating *Formica armata* (Le Guillou 1842) as its type species. While Forel did not provide a definition, the subgenus was later delimited by Emery (1925). By that time, *Myrmhopla* already encompassed approximately 140 nomina, prompting Emery to subdivide it into 6 species-groups to manage its considerable diversity. This framework was significantly expanded by Dorow (1995), who recognized a total of 16 species-groups by adding 10 new ones to the original 6. In terms of natural history, *Myrmhopla* species are known for their remarkable nesting habits, including arboreal carton nests and nests in bamboo internodes. Many species exhibit strong associations with specific host plants, such as bamboos, and their larvae produce silk used to bind nest materials (Dorow 1995, Robson and Kohout 2007). The strongly developed propodeal and petiolar spines in this subgenus are thought to function as defensive adaptations against predators.

The study of the ant genus *Polyrhachis* in China began with Smith (1858), who described the first 2 species from Hong Kong and southern China. Subsequently, numerous new species have been described by successive authors (Forel 1879, Wang and Wu 1991, Kohout 1994, Wu and Wang 1995, Zhou and Zheng 1997, Xu 1998, 2002a, Zhou and Huang 2002, Qian and Zhou 2008, Wong and Guénard 2020, Chen et al. 2025b) more than 20 species. To date, a total of 56 species of *Polyrhachis* have been recorded in China (AntWiki 2025, Chen et al.2025b).

This work contributes to the taxonomy of the genus *Polyrhachis* in China by describing 2 new species from southern China under the subgenera *Myrma* and *Myrmhopla* and by providing comprehensive keys for the identification of all Chinese species in these 2 subgenera.

## Materials and Methods

The ant specimens were obtained using sample plots and search-collecting methods (eg Xu 2002b). Sample plots were set up at the research site with different altitude gradients and included a variety of vegetation types within the survey area. Subsequently, the specimens were examined using a Nikon SMZ 745 stereomicroscope. High-quality multifocus montage images were captured using a Keyence VHX-6000 ultra-depth microscopic three-dimensional microscope, and the images were combined into graphics using Adobe Photoshop 2020. Morphological line drawings were produced using Procreate software (version 5.4.7, Savage Interactive Pty Ltd., Hobart, Tasmania, Australia). To compare the worker morphology of the 2 new species, reference was made to the original descriptions of related species (Dorow et al. 1997, Kohout 2006, 2007, 2008a, 2008b, 2010, 2012, 2013, Xu 1998, 2002a, Wong and Guénard 2020). The keys were prepared using the examined specimens, images available on AntWeb (2025) and AntWiki (2025), and original descriptions of the species. The holotype images of *Polyrhachis fellowesi*, *Polyrhachis hunggeuk*, *Polyrhachis confusa*, and *Polyrhachis peetersi* are reproduced from Wong and Guénard (2020). The line drawings of *Polyrhachis tianjingshanensis*, *Polyrhachis jianghuaensis*, *Polyrhachis zhengi*, *Polyrhachis euthiacaena*, and *Polyrhachis rubigastrica* are digitally traced and refined from the original illustrations in their respective protologues.

The investigated material is deposited in the following institutions:

“KIZ”—Kunming Natural History Museum of Zoology, Kunming Institute of Zoology, Chinese Academy of Sciences, Kunming, Yunnan Province, China.

“SWFU”—Insect Collection, Southwest Forestry University, Kunming, Yunnan Province, China.

**Fig. 1.**
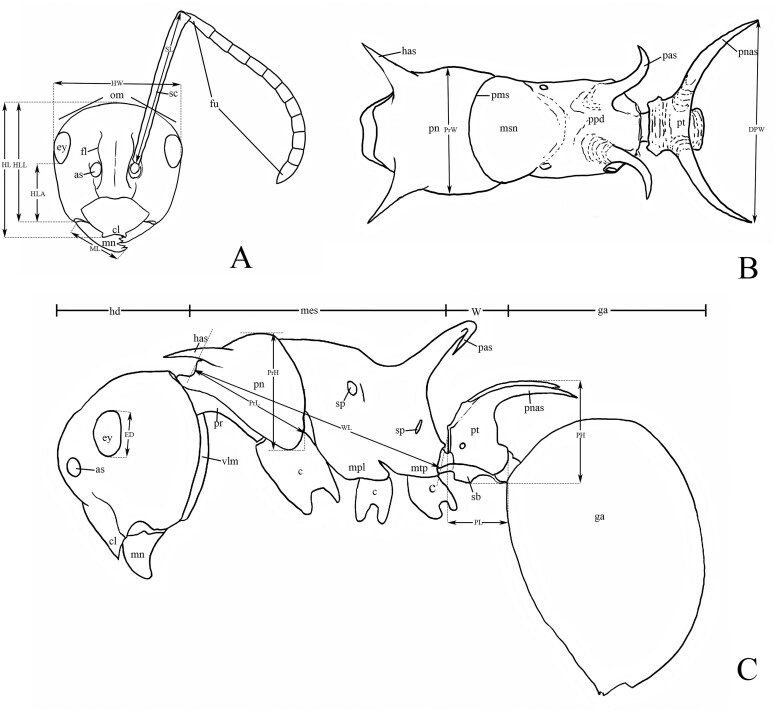
Line drawing and measurements of worker of *Polyrhachis* species. (A) Head in full‑face view; (B) body in dorsal view; (C) body in lateral view. as, antennal socket; c, coxa; cl, clypeus; ey, eye; fl, frontal lobe; fu, funiculus; ga, gaster; has, humeri armed spine; hd, head; mes, mesosoma; mn, mandible; mpl, mesopleuron; msn, mesonotal; mtp, metapleuron; om, occipital margin; pas, propodeum armed spine; pms, promesonotal suture; pn, pronotum; pnas, petiolar node armed spines; ppd, propodeum; pr, propleuron; pt, petiole; sb, subpetiolar process; sc, scape; sp, spiracle; vlm, ­ventrolateral margin; W, waist.

Standard measurements and indices were employed as defined in Bolton (1975), Wong and Guénard (2020), and Chen et al. (2025a). All measurements are expressed in millimeters (Fig. 1).

“HL”—Head length: the straight-line length of the head in perfect full-face view, measured from the midpoint of the anterior clypeal margin to the midpoint of the posterior margin. In species where 1 or both of these margins are concave, the measurement is taken from the mid-point of a transverse line that spans the apices of the projecting portions.

“HLL”—Head lateral margin length: in full-face view, the head length measured from the mandible base to the nuchal carina.

“HLA”—Anterior head length: in full-face view, the head length measured from the mandible base to the anterior edge of the eye.

“HW”—Head width: the maximum width of the head in full-face view, excluding the compound eyes.

“ML”—Mandible length: the straight-line length of the mandible measured from the apex to the lateral base.

“SL”—Scape length: the straight-line length of the antennal scape, excluding the basal constriction or neck.

“ED”—Eye diameter: the maximum diameter of the compound eye.

“PrL”—Pronotum length: in profile, the diagonal length of the pronotum, measured from the anterior margin of the pronotum excluding the collar to the posterior extremity of the pronotum.

“PrH”—Pronotum height: in profile, the maximum height of the pronotum, measured from the posterior base of the lateral margin of the pronotum to the highest point of the pronotum.

“PrW”—Pronotum width: the maximum width of the pronotum measured in dorsal view.

“WL”—Weber’s length (=alitrunk length): the diagonal length of the mesosoma in lateral view, measured from the point at which the pronotum meets the cervical shield to the posterior basal angle of the metapleuron.

“TL”—Total length: the total outstretched length of the individual, from the mandibular apex to the metasomal apex (ie the posteriormost point of the gaster, which in *Polyrhachis* comprises the third abdominal segment (abdominal tergite III) onwards, excluding the petiole).

“PL”—Petiole length: the length of the petiole measured in lateral view from the anterior process to the posteriormost point of the tergite, where it surrounds the helcium.

“PH”—Petiole height: the height of the petiole measured in lateral view from the apex of the ventral (subpetiolar) process vertically to a line intersecting the dorsal most point of the node.

“DPW”—Dorsal petiole width: the maximum width of the petiole in dorsal view.

“CI”—Cephalic Index = HW × 100/HL.

“SI”—Scape index = SL × 100/HW.

“EI”—Eye Index= ED × 100/HW.

“MI”—Mandible Index= ML × 100/HL.

“LPI”—Lateral petiole index = PH × 100/PL.

“PDPI”—Dorsal petiole index = DPW × 100/PL.

Below, a drawing showing an entire ant with various morphological features that are mentioned in the text labeled (Gu et al. 2025) (Fig. 1).

### Taxonomic Results

Genus ***Polyrhachis***  Smith 1857

*Polyrhachis*  Smith 1857: 58. Type species: *Formica bihamata* Drury 1773, by original designation.

Subgenus ***Myrma* Billberg 1820** 

*Myrma* Billberg 1820: 104 Type-species: *Formica militaris* (obsolete combination of *Polyrhachis militaris*), by subsequent designation of Wheeler 1911: 859.

#### *Polyrhachis aureodorsalis* sp. nov. Chen 2026

https://zoobank.org/act:909FFFD5-1FA5-478A-8CDF-A13617C10C31

**Fig. 2.**
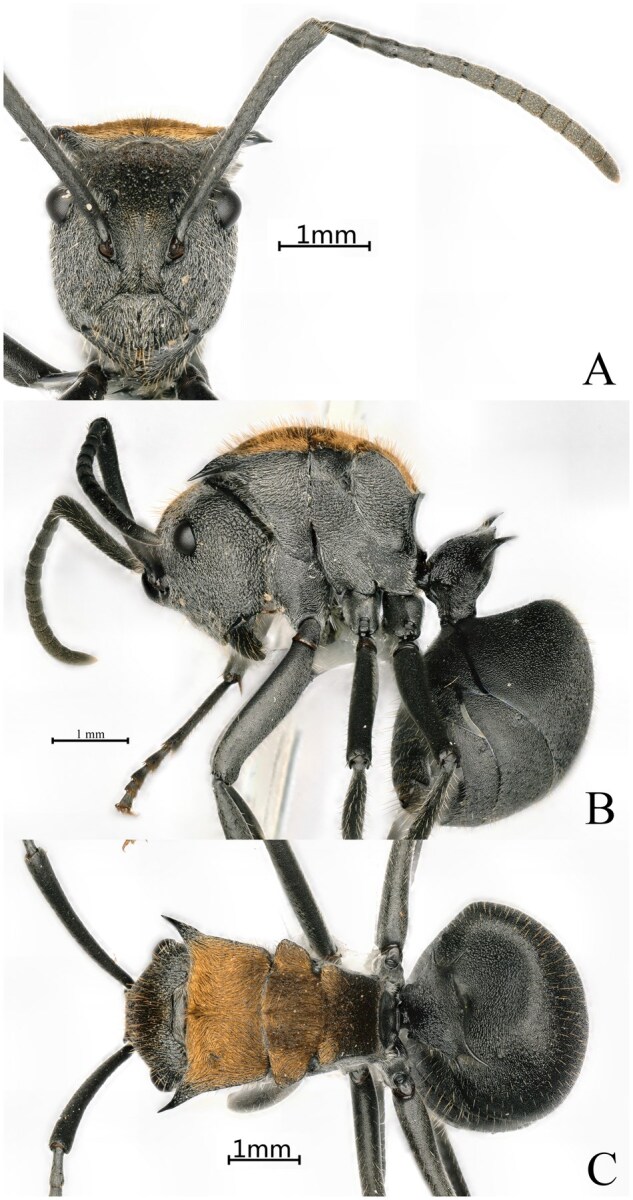
*Polyrhachis aureodorsalis* **sp. nov**. worker (Holotype, No. S20250068, imaged by Chao Chen). (A) Head in full-face view; (B) body in lateral view; (C) body in dorsal view.

**Fig. 3.**
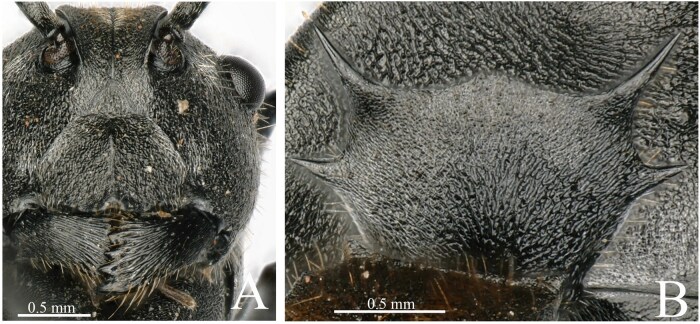
*Polyrhachis aureodorsalis* **sp. nov**. worker (Holotype, No. S20250068, imaged by Chao Chen). (A) Mandibles in full-face view; (B) petiole in frontal view.

Figs 2A–C and 3A and B

##### Etymology

The species name is derived from the Latin “aureus” (golden) and “dorsalis” (pertaining to the back), alluding to the characteristic golden hair and pubescence on the dorsal surface of the mesosoma.

##### Type material

***Holotype*:** worker, **China**: Fujian Province, Sanming City, Youxi County, Xinyang Town, 26.11445 N, 118.06591 E, 533 m above sea level, from ground in mixed coniferous and broad-leaved forest, 25.vi.2025, leg. Longguan Lin, No.S20250068 (KIZ). ***Paratypes*:** 4 workers, same data as holotype. S20250068A and S20250068B (SWFU); S20250068C and S20250068D (KIZ).

##### Description

**Holotype worker:** HL 2.27; HLL 2.08; HLA 1.14; HW 2.05; ML 0.91; SL 2.89; ED 0.48; PrL 1.38; PrH 1.86; PrW 2.19; WL 3.35; TL 10.45; PL 1.01; PH 1.50; DPW 1.85; CI 90; SI 141; EI 23; MI 40; LPI 149; and PDPI 184. **Paratype workers:** (*n* = 4) (Supplementary Table S1): HL 2.18 to 2.24; HLL 2.05 to 2.12; HLA 1.08 to 1.16; HW 1.93 to 2.06; ML 0.83 to 0.89; SL 2.75 to 2.96; ED 0.44 to 0.49; PrL 1.10 to 1.39; PrH 1.74 to 1.83; PrW 1.99 to 2.21; WL 2.99 to 3.26; TL 9.75 to 10.60; PL 0.87 to 0.97; PH 1.50 to 1.55; DPW 1.77 to 1.84; CI 84 to 95; SI 138 to 149; EI 23 to 25; MI 37 to 39; LPI 158 to 177; and PDPI 188 to 205.

In full-face view, head oval-shaped, slightly longer than broad (CI 90). Lateral margins in front of compound eyes slightly convex, converging anteriorly toward mandibular bases; posteriorly, occipital margin strongly biconvex with a distinct median emargination. Compound eyes situated in posterior half of head, facing laterally; moderately convex, distinctly exceeding lateral cephalic outline. Frontal carinae sinuate and laminate, obscuring approximately the dorsal one-third of the antennal sockets. Clypeus with a distinct median carina; its anterior margin arcuate with a median notch, posterior margin almost straight with a slight median emargination, merging with the impressed basal margin. Mandibles with masticatory margin bearing 5 teeth, apical tooth longest, subsequent teeth gradually reducing in size (Fig. 3A). Antenna with 12 antennomeres; scape robust, much longer than head width (SI 141); antennal segment II (pedicel) longer than subsequent funicular segments (Fig. 2A).

In lateral view, posterior margin of compound eye slightly flattened, ventrolateral margin of head angular. Mesosomal dorsum distinctly marginate and prominently arched in profile, reaching its highest point at promesonotal suture. Pronotal sides triangular; humeri angular, armed with spines directed anteriorly. Promesonotal suture distinct and deeply impressed. Mesometapleural suture distinct and deeply impressed, connecting to spiracle laterally. Dorsum of propodeum and the declivity are separated by a pair of upwardly curved teeth arising from posterodorsal margin of propodeum; declivity concave. Metapleural spiracle slit-shaped. Petiole triangular in overall shape, narrower dorsally and broader ventrally. In profile, both anterior and posterior margins of petiole moderately convex, while ventral margin sinuate with a median projection. Gaster oval; anterior margin concave, and both tergites and sternites evenly arched (Fig. 2B).

In dorsal view, pronotum widest across its anterior lateral angles; lateral margins straight and converge posteriorly, anterior margin sinuate, and pronotal spines directed anterolaterally. Mesonotal lateral margins strongly convex and laminate. Pomesonotal suture distinct. Mesometapleural suture distinct. Propodeum has obtusely angular anterior lateral corners and bears a pair of small denticles on its posterior lateral margins. The lateral margins of promesonotum and propodeum strongly constricted at their junction. Petiole armed with 2 pairs of sharp spines, it bears a pair of small lateral spines and a pair of longer, upwardly directed dorsal spines, and its dorsal margin moderately convex medially (Fig. 3B). Anterior face of first gastral tergite concave, extensively rounding onto dorsum of segment (Fig. 2C).

***Pilosity.*** Head in full-face view with a medium density of white, appressed pilosity; anterior portion of head with a medium density of yellowish, erect setae, and posterior portion with a medium density of yellow, erect setae. Scapes with sparse, short, white, erect hairs. Mesosoma laterally, propodeal declivity, and fore coxae with a dense covering of white, appressed pilosity; mesosomal dorsum bears dense, golden, appressed pilosity intermixed with dense, golden, erect setae. Dorsal and ventral surfaces of gaster, as well as tibiae, bear sparse, yellowish, erect setae. Inner surface of tarsi covered with dense, golden, suberect hairs.

***Sculpture.*** Mandibles longitudinally striate, with a row of piliferous pits along the dorsal masticatory margin and anterior clypeal margin. Head, mesosoma, and petiole costulate. Gaster densely punctate. Legs imbricate.

***Coloration.*** Body entirely black.

##### Comparative notes

The new species is similar to *P. fellowesi* Wong and Guénard. In full-face view of *Polyrhachis aureodorsalis* **sp. nov.**, head slightly longer than broad (CI 90), posteriorly, occipital margin strongly biconvex with a distinct median emargination; frontal carinae widely separated. In lateral view, humeri angular relatively thick; mesosomal dorsum bears dense, golden, appressed pilosity intermixed with dense, golden, erect setae. Conversely, in full-face view of *P. fellowesi*, head clearly longer than broad (CI 70 to 75), posteriorly, occipital margin strongly convex; frontal carinae narrowly separated. In lateral view, humeri angular relatively thinner; mesosomal dorsum bears pale yellow, appressed pilosity intermixed with sparse, whitish, erect setae.

##### Distribution

China (Fujian).

##### Habitat

**Fig. 4.**
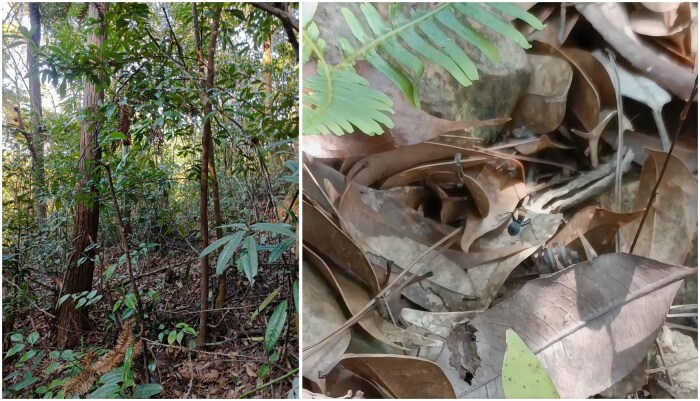
Collection sites of *Polyrhachis aureodorsalis* **sp. nov**.

This new species inhabits mixed coniferous-broadleaf forests dominated by *Quercus* spp. and *Pinus* spp. The type specimens were collected foraging on ground litter in an open area at 14:30, with a recorded ambient temperature of 32 °C (Fig. 4).

#### Key to species of the subgenus *Myrma* found in China based on the worker caste

The identifying characteristics of some species in this key are based on Wong and Guénard (2020) and Kohout (2013).

**Fig. 5.**
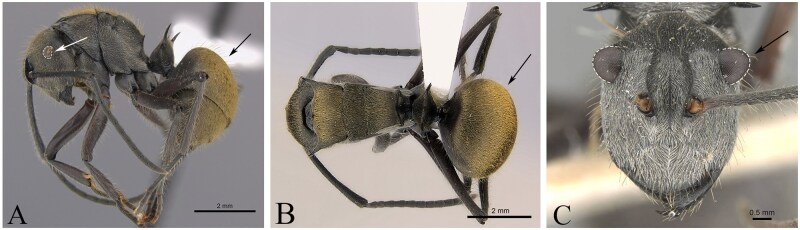
*Polyrhachis vigilans* worker (CASENT227850, non-type). (A) Body in lateral view; (B) body in dorsal view; (C) head in full-face. Photographer: Michele Esposito.

**Fig. 6.**
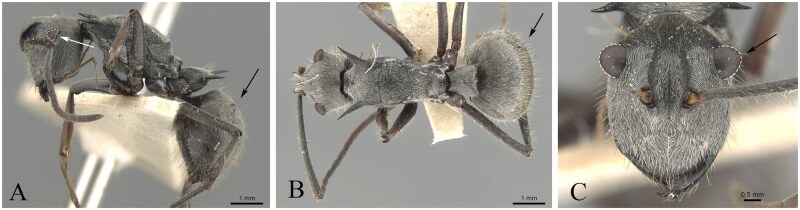
*Polyrhachis pubescens* worker (CASENT0906795, non-type). (A) Body in lateral view; (B) body in dorsal view; (C) head in full-face. Photographer: Michele Esposito.

**Fig. 7.**
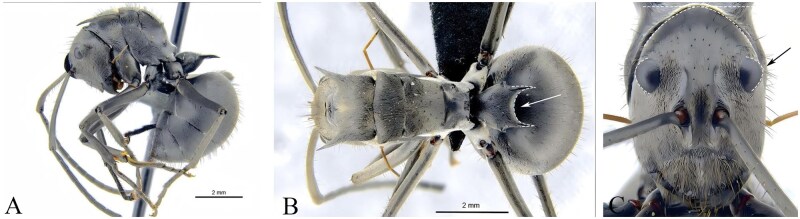
*Polyrhachis fellowesi* worker (WTL0007, type). (A) Body in lateral view; (B) body in dorsal view; (C) head in full-face. (Images cited from Wong and Guénard 2020).

**Fig. 8.**
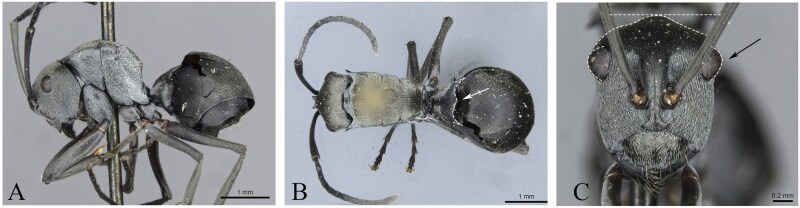
*Polyrhachis latona* worker (CASENT0910920, type). (A) Body in lateral view; (B) body in dorsal view; (C) head in full-face. Photographer: Z. Lieberman.

**Fig. 9.**
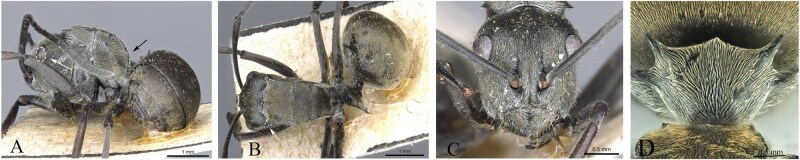
*Polyrhachis murina* worker (CASENT0905612, type). (A) Body in lateral view; (B) body in dorsal view; (C) head in full-face; (D) petiole in frontal view (cited from Kohout 2013, fig 41). Photographer: Z. Lieberman.

**Fig. 10.**
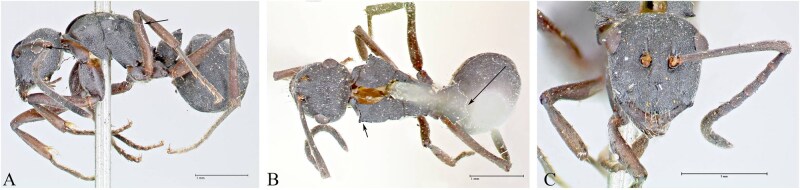
*Polyrhachis punctillata* worker (FOCOL2622, type). (A) Body in lateral view; (B) body in dorsal view; (C) head in full-face. Photographer: Christiana Klingenberg.

1. In lateral view, compound eyes strongly posteriorly truncate (Figs 5A and 6A)………………………………………………….2

- In lateral view, compound eyes normal or only weakly posteriorly truncate (Figs 9A and 10A)………………………………5

2. (1) In full-face view, compound eyes strongly convex, with approximately half of their length exceeding lateral cephalic outline (Figs 5C and 6C)……………………………………….3

- In full-face view, compound eyes weakly to moderately convex, slightly exceeding lateral cephalic outline (Figs 7C and 8C)……………………………………………………………….4

3. (2) TL ≥ 10 mm (Fig. 5A); CI 84 to 96 (Fig. 5C); gaster with golden pubescence (Fig. 5B)……………………………………………………………………………. *Polyrhachis vigilans* Smith

- TL < 10 mm (Fig. 6A); CI 75 to 79 (Fig. 6C); gaster with whitish to pale yellowish pubescence (Fig. 6B)…………………………. *Polyrhachis pubescens* Mayr

4. (2) TL ≥ 10 mm (Fig. 7A); head in full-face view posterior margin shallowly and roundly convex (Fig. 7C); petiole seen from back dorsal margin almost flat; body extensively covered with pale to dark brown erected setae (Fig. 7B)…………………………………………………*Polyrhachis fellowesi* Wong and Guénard

- TL < 10 mm (Fig. 8A); head in full-face view posterior margin shallowly convex (Fig. 8C); petiole seen from back dorsal margin convex medially, sometimes forming blunt to distinct median tooth; erect setae predominantly confined to clypeus, abdominal sternites, and gastral apex (Fig. 8B); in some specimens, a few erect setae may also be present on the frons near the epistomal sulcus……………. *Polyrhachis latona* Wheeler

5. (1) In dorsal view, pronotum armed with triangular teeth or short spines (Figs 9B and 10B)…………………………………….6

- In dorsal view, pronotum armed with long spines on its anterolateral corners (Figs 12B and 13B)……………………….8

6. (5) In profile, propodeum bears reduced, indistinct spines (Fig. 9A). In dorsal view, dorsal margin of petiole convex medially (Fig. 9D)……………………………………………………………………………………. *Polyrhachis murina* Emery

- In profile, propodeum armed with distinct, triangular, denticulate spines (Fig. 10A). In dorsal view, dorsal margin of petiole concave medially (Fig. 10B)……………….………….7

7. (6) Petiolar node armed with 2 dorsal and 2 lateral teeth, all nearly equal in both distance and size. Propodeal dorsum bears sparse erect setae (Fig. 10)………………………………………………………………………… *Polyrhachis punctillata* Roger

- Apex of petiolar node unarmed, appearing rounded or at most with obsolete denticles. Propodeal dorsum bears abundant erect setae (Fig. 11)…………………………*P. subpilosa* Emery

**Fig. 11.**
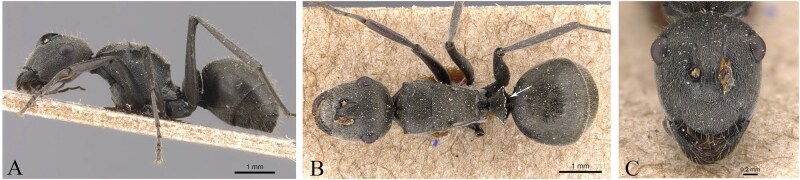
*Polyrhachis subpilosa* worker (CASENT0905830, type). (A) Body in lateral view; (B) body in dorsal view; (C) head in full-face. Photographer: Will Ericson.

**Fig. 12.**
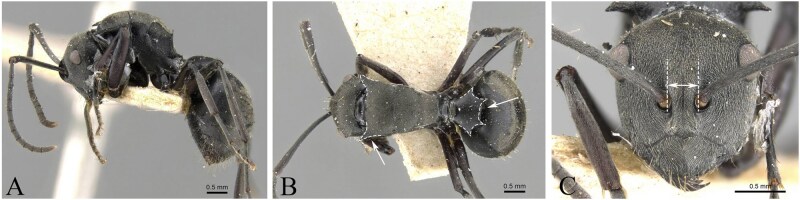
*Polyrhachis convexa* worker (CASENT0906801, non-type). (A) Body in lateral view; (B) body in dorsal view; (C) head in full-face. Photographer: Michele Esposito.

**Fig. 13.**
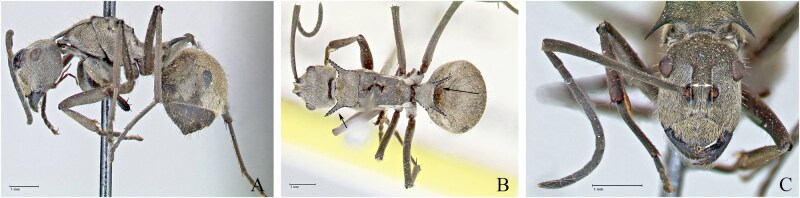
*Polyrhachis proxima* worker (FOCOL2624, type). (A) Body in lateral view; (B) body in dorsal view; (C) head in full-face. Photographer: Christiana Klingenberg.

**Fig. 14.**
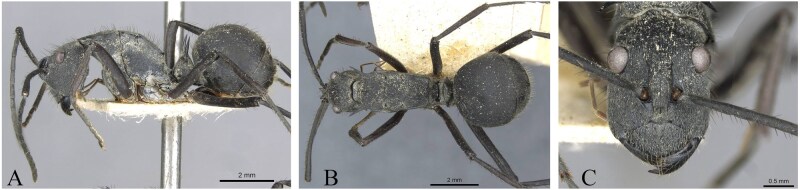
*Polyrhachis striata* worker (CASENT0910928, type). (A) Body in lateral view; (B) body in dorsal view; (C) head in full-face. Photographer: Z. Lieberman.

**Fig. 15.**
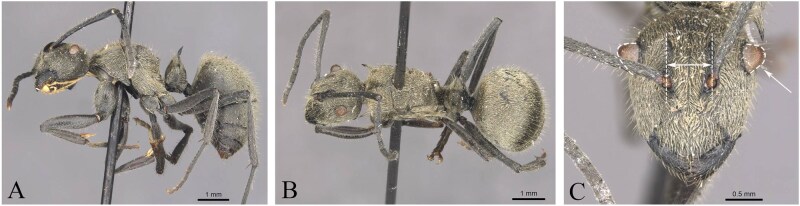
*Polyrhachis wolfi* worker (CASENT0910937, type). (A) Body in lateral view; (B) body in dorsal view; (C) head in full-face. Photographer: Will Ericson.

**Fig. 16.**
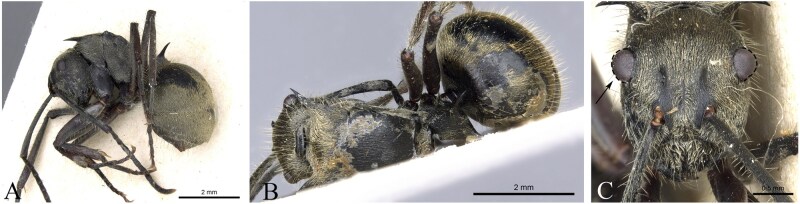
*Polyrhachis illaudata* worker (CASENT0903443, type). (A) Body in lateral view; (B) body in dorsal view; (C) head in full-face. Photographer: Z. Lieberman.

8. (5) Frontal carinae narrowly separated, with intercarinal distance at its widest point ≤0.5 mm (Figs 12C and 13C)……………………………………………………………….9

- Frontal carinae widely separated, with intercarinal distance at its widest point >0.5 mm (Figs 2A and 15C)……………………………………………………………….11

9. (8) Head elliptical in full-face view, with lateral margins converging anteriorly from compound eyes toward mandibular bases (Fig. 12C). Petiolar node armed with 2 dorsal and 2 lateral teeth, all nearly equidistant and subequal in size (Fig. 12B)………………………… *Polyrhachis convexa* Roger

- Head trapezoidal in full-face view, with lateral margins diverging anteriorly from compound eyes toward mandibular bases (Fig. 13C). Petiolar node armed with 2 dorsal teeth and 2 lateral teeth; the dorsal pair is markedly larger in size and exhibits the greatest interdenticular distance (Fig. 13B)……………..………………………………………10

10. (9) Body densely pubescent; mesosoma laterally lacking long striae (Fig. 13)……………………………………………………………………………………. *Polyrhachis proxima* Roger

- Body with very sparse pubescence; mesosoma laterally with distinct long striae (Fig. 14)………………………………………………………………………………. *Polyrhachis striata* Mayr

11. (8) Head and lateral mesosoma with a medium density of whitish pubescence. Mesosomal dorsum with a dense covering of golden pubescence intermixed with dense, golden, erect setae (Fig. 2)………………….*Polyrhachis aureodorsalis* **sp. nov.** Chen

- Body densely covered with whitish to pale yellowish pubescence, overlain by a medium density of whitish to pale yellowish, erect setae (Figs 15 and 16)……………………………….12

12.(11) In full-face view, compound eyes more convex, distinctly narrowed distally (Fig. 15C)……*Polyrhachis wolfi* Forel

**Fig. 17.**
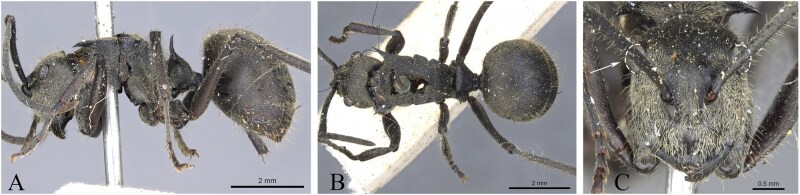
*Polyrhachis tyrannica* worker (CASENT0903442, type). (A) Body in lateral view; (B) body in dorsal view; (C) head in full-face. Photographer: Z. Lieberman.

- In full-face view, compound eyes less convex, curvature surface more rounded (Figs 16C and 17C)…………………….13

13. (12) Golden yellow appressed pilosity distributed over most body surfaces; femora and tibiae black (Fig. 16)………………………… *Polyrhachis illaudata* Walker

- Pale yellow appressed pilosity distributed over most body surfaces; femora and tibiae reddish brown (Fig. 17)…………………………. *Polyrhachis tyrannica* Smith

#### Subgenus *Myrmhopla* Forel 1915

*Myrmhopla* Forel 1915b: 107 (as subgenus of *Polyrhachis*). Type-species: *Formica armata* (obsolete combination of *Polyrhachis armata*), by original designation.

#### *Polyrhachis mangensis* sp. nov. Chen 2026

**Fig. 18.**
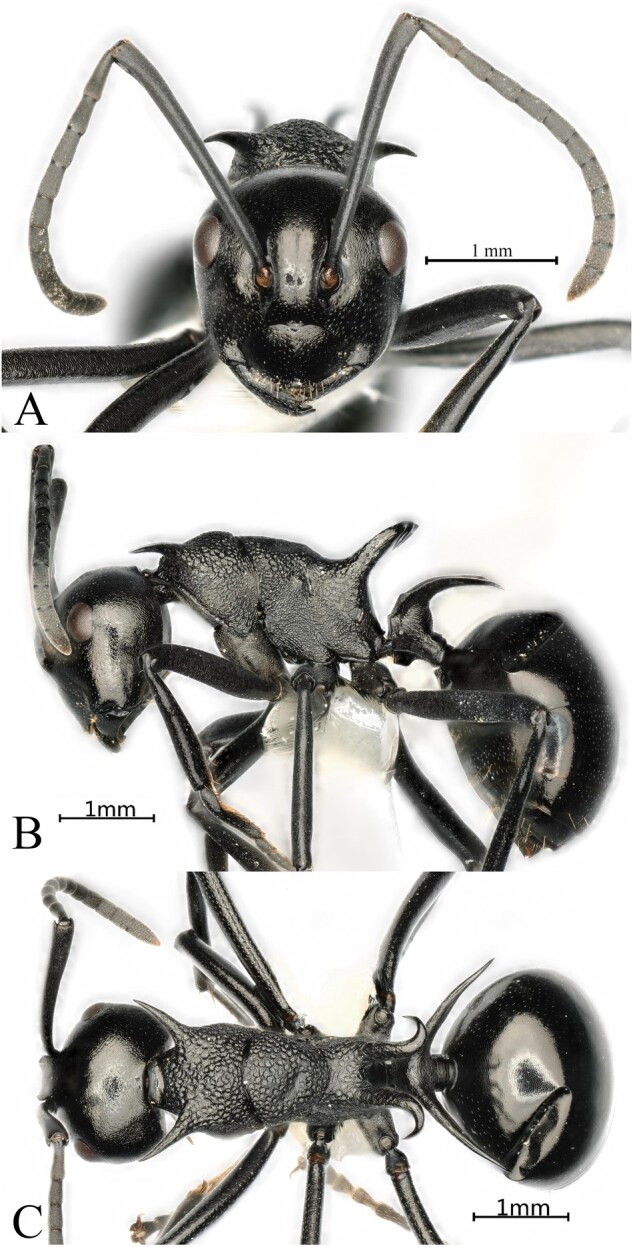
*Polyrhachis mangensis* **sp. nov**. worker (Holotype, No. S20250012, imaged by Chao Chen). (A) Head in full-face view; (B) body in lateral view; (C) body in dorsal view.

**Fig. 19.**
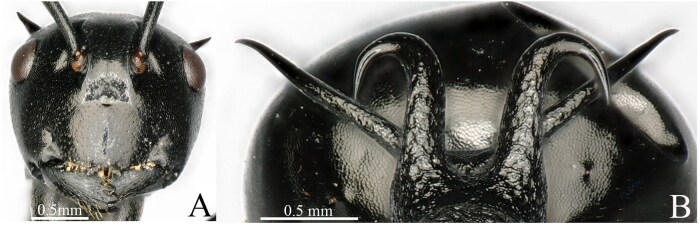
*Polyrhachis mangensis* **sp. nov**. worker (Holotype, No. S20250012, imaged by Chao Chen). (A) Mandibles in full-face view; (B) petiole in frontal view.

https://zoobank.org/act:9483B945-782A-4257-AE2A-36D39660B512

(Figs 18A–C and 19A and B)

##### Etymology

The specific epithet mangensis is a modern Latin adjective derived from “Mang,” the name of the city in Dehong Prefecture, Yunnan Province, China, where the type series was collected.

##### Type material

***Holotype*:** worker, **China**: Yunnan Province, Dehong Dai and Jingpo Autonomous Prefecture, Mang City, Laxiang Village, 24.20383 N, 98.17698 E, 816 m above sea level, from bamboo leaf in bamboo forest, 7.vii.2025, leg. Chao Chen, No. S20250012 (KIZ). ***Paratypes*:** 3 workers, same data as holotype. S20250012A (SWFU); S20250012B; and S20250012C (KIZ).

##### Description

**Holotype workers:** HL 1.76; HLL 1.62; HLA 0.74; HW 1.69; ML 0.74; SL 2.09; ED 0.45; PrL 1.06; PrH 1.11; PrW 1.22; WL 2.52; TL 7.82; PL 0.57; PH 1.20; DPW 2.26; CI 96; SI 124; EI 27; MI 42; LPI 212; and PDPI 399. **Paratype workers:** (*n* = 3) (Supplementary Table S2): HL 1.70 to 1.76; HLL 1.54 to 1.61; HLA 0.75 to 0.77; HW 1.65 to 1.69; ML 0.72 to 0.73; SL 2.01 to 2.09; ED 0.44 to 0.46; PrL 0.99 to 1.05; PrH 1.08 to 1.11; PrW 1.16 to 1.18; WL 2.40 to 2.62; TL 7.39 to 7.78; PL 0.51 to 0.54; PH 1.17 to 1.21; DPW 1.81 to 2.10; CI 95 to 97; SI 122 to 124; EI 27; MI 41 to 43; LPI 218 to 238; and PDPI 356 to 392.

In full-face view, head elliptical, slightly longer than broad (CI 96), with lateral margins strongly convex, converging anteriorly toward mandibular bases; occipital margin moderately convex. Compound eyes situated in posterior half of head, facing laterally; weakly convex, not or only slightly exceeding lateral cephalic outline. Mandibles triangular, with base distinctly concave; masticatory margin with 5 teeth, apical tooth longest, subsequent teeth gradually reducing in size. Clypeus evenly convex, without a distinct median carina; its anterior margin with a distinct median notch, flanked by small denticles on either side; posterior margin sinuate (Fig. 19A). Frontal carinae sinuate, extending posteriorly to the level of the posterior compound eye margin, not obscuring antennal sockets. Antenna with 12 antennomeres; scape long, gradually thickening from base to apex, much longer than head width (SI 124); antennal segment II (pedicel) longer than subsequent funicular segments (Fig. 18A).

In lateral view, mandibles with base distinctly concave; ventrolateral margin of head evenly convex, forming a continuous arc. Pronotal dorsum flat medially, rising posteriorly to form highest point of mesosoma, armed anteromedially with a pair of anteriorly directed spines. Promesonotal suture distinct and deeply impressed; mesonotal dorsum strongly convex medially. Mesopleural spiracle semilunate in outline. Propodeum armed at junction of dorsal face and declivity with a pair of long spines, apical one-quarter of each spine curved through 180°, forming a distinct hook; propodeal declivity nearly straight; propodeal spiracle slit-shaped. Petiole trapezoidal in overall outline; its dorsal margin armed apically with a pair of posteriorly directed long spines; the anterior margin of node with a pair of weak denticles medially, posterior margin moderately convex; subpetiolar process subrectangular, ventral margin straight. Gaster oval; anterior margin almost straight; gastral tergites and sternites evenly arched Anterior face of first gastral tergite convex, extensively rounding onto dorsum of segment (Fig. 18B).

In dorsal view, scape bent at the basal one-quarter. Pronotum with lateral margins strongly convex, anterior and posterior margins emarginate; anterolateral corners each armed with a spine directed anterolaterally. Promesonotal suture distinct and deeply impressed. Mesonotal lateral margins concave, anterior and posterior margins convex. Propodeum armed with a pair of long, robust spines; each spine robust at base, acutely pointed at tip, initially subparallel before curving through 180° and diverging laterally at their apices. Petiolar node armed apically with a pair of long, V-shaped spines directed posterolaterally (Fig. 18C).

***Pilosity.*** Head in full-face view, antennal funiculi, tibiae, and gastral tergites and sternites with a medium density of minute, whitish, appressed hairs. Protarsal segments with yellowish pubescence intermixed with short, suberect hairs. Anterior clypeal margin, external mandibular margins, anteroventral margin of petiole, and gastral sternites with sparse, yellowish, erect setae.

***Sculpture.*** Mandibles finely longitudinally striate, with piliferous pits along the dorsal masticatory margin. Head in full-face view, legs and gaster finely and densely shagreened. Promesonotal dorsum densely and moderately punctate. Lateral mesosoma and sides of petiole densely and transversely punctate. Propodeal and petiolar spines smooth and shining at their apices.

***Colouration.*** Body entirely black.

##### Comparative notes

The new species is similar to *Polyrhachis arachne* Emery. In full-face view of the new species, anterior clypeal margin has 2 closely spaced denticles with a distinct median emargination; pronotal spines short and directed anteriorly; posterior part of pronotal dorsum moderately convex; lateral propodeum costulate; propodeal spines robust at their base; body entirely black. Conversely, in full-face view of *P. arachne*, anterior clypeal margin has 2 widely separated denticles and median emargination nearly absent, appearing almost straight; pronotal spines long and directed anterodorsally; posterior pronotal dorsum strongly convex; lateral propodeum densely punctate; propodeal spines comparatively narrower at base; head, mesosoma, and propodeum brownish, with reddish-brown legs and gaster.

##### Distribution

China (Yunnan).

##### Habitat

**Fig. 20.**
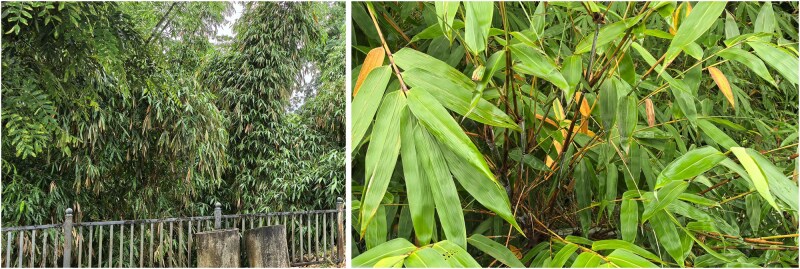
Collection sites of *Polyrhachis mangensis* **sp. nov**.

This new species inhabits bamboo forests (*Dendrocalamus latiflorus* Munro). The type specimens were collected foraging on bamboo leaves at 10:00, under light rain and an ambient temperature of 21 °C (Fig. 20).

#### Key to species of the subgenus *Myrmhopla* found in China based on the worker caste

The following key to the known Chinese species of the subgenus *Myrmhopla* is modified from Dorow (1995) framework for these species-groups, incorporating the newly described species. *Polyrhachis tschu* Forel is excluded from this key as it is known only from the queen caste. The identifying characteristics of some species in this key are based on Wong and Guénard (2020).

**Fig. 21.**
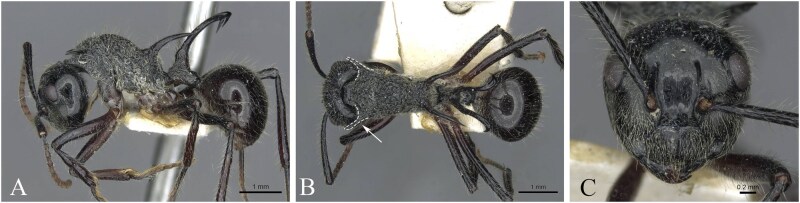
*Polyrhachis furcata* worker (CASENT0910867, type). (A) Body in lateral view; (B) body in dorsal view; (C) head in full-face. Photographer: Z. Lieberman.

**Fig. 22.**
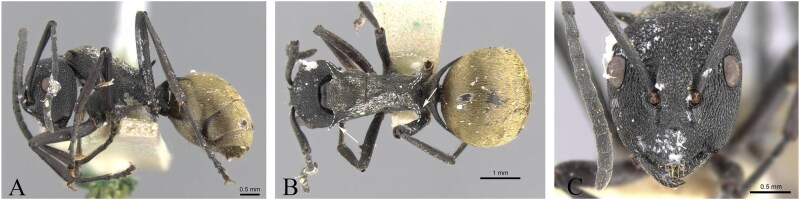
*Polyrhachis rupicapra* worker (CASENT0906783, non-type). (A) Body in lateral view; (B) body in dorsal view; (C) head in full-face. Photographer: Michele Esposito.

**Fig. 23.**
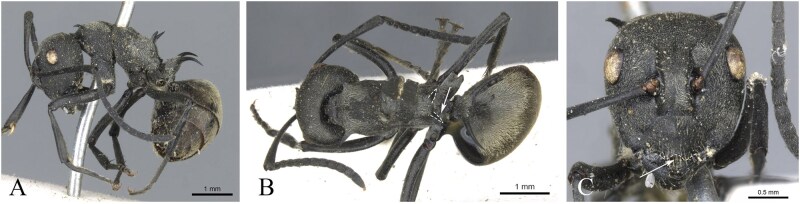
*Polyrhachis dives* worker (CASENT0903388, type). (A) Body in lateral view; (B) body in dorsal view; (C) head in full-face. Photographer: Will Ericson.

**Fig. 24.**
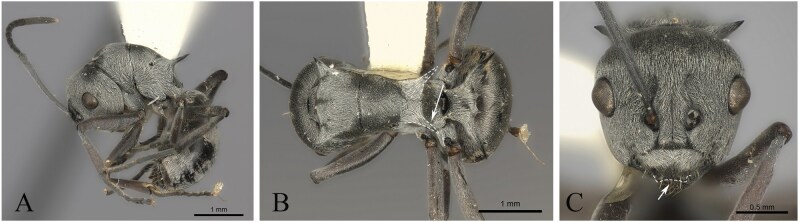
*Polyrhachis tibialis* worker (CASENT0217805, non-type). (A) Body in lateral view; (B) body in dorsal view; (C) head in full-face. Photographer: Michele Esposito.

**Fig. 25.**
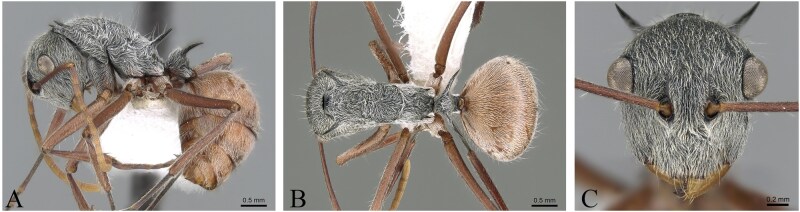
*Polyrhachis bicolor* worker (CASENT021406, non-type). (A) Body in lateral view; (B) body in dorsal view; (C) head in full-face. Photographer: Estella Ortega.

**Fig. 26.**
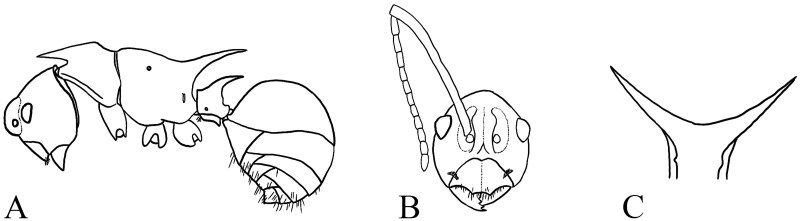
*Polyrhachis tianjingshanensis* worker (type). (A) Body in lateral view; (B) Head in full-face; (C) petiole in frontal view. (Line drawing cited from Qian and Zhou 2008).

1. In dorsal view, humeri armed with conspicuous, long, and spinose spines (Figs 21 and 22)………………………………….2

- In dorsal view, humeri bluntly angular, at most with small denticles (Figs 34 and 35)………………………………………14

2. (1) Mesosomal dorsum with medium-density to dense suberect and appressed pilosity (Figs 21 and 22)………………….3

- Mesosomal dorsum hairless or with only sparse erect setae (Figs 18 and 28)………………………………………………….9

3. (2) Petiolar spines exceptionally long, their apical portion uncinate (hook-like) (Fig. 21)………………………………………………………………………………*Polyrhachis furcata* Smith

- Petiolar spines moderate or short in length, their tips acutely pointed, not uncinate (Figs 22 and 23)……………………….4

4. (3) Body uniformly black to dark brown (Figs 22 and 23)…………………………………………………………………5

- Head, mesosoma, and petiole black, gaster reddish to brown (Figs 25 and 26)………………………………………………….7

5. (4) Propodeal spines strongly curved laterally at their apices (hook-like in dorsal view) (Fig. 22B)………………………………………………………………….*Polyrhachis rupicapra* Roger

- Propodeal spines straight or only weakly curved, directed posterolaterally (Figs 23B and 24B)…………………………….6

6. (5) Anterior clypeal margin with a median emargination between 2 denticles (Fig. 23C); propodeal spines in lateral view shorter than length of propodeal declivity; petiolar spines arising from lateral margin below the dorsal margin and directed posteriorly (Fig. 23A)……………………………………………………………………………………….*Polyrhachis dives* Smith

- Anterior clypeal margin nearly straight between 2 denticles (Fig. 24C); propodeal spines in lateral view equal to or longer than length of propodeal declivity; petiolar spines arising from dorsal margin and directed upward (Fig. 24A)……………………………………………………………*Polyrhachis tibialis* Smith

7. (4) Antennae, mandibles, and legs rufous; body with abundant whitish pubescence (Fig. 25)…………………………………………………………………………*Polyrhachis bicolor* Smith

- Antennae, mandibles, and legs black; body with sparse whitish pubescence (Figs 26 and 27)………………………………8

**Fig. 27.**
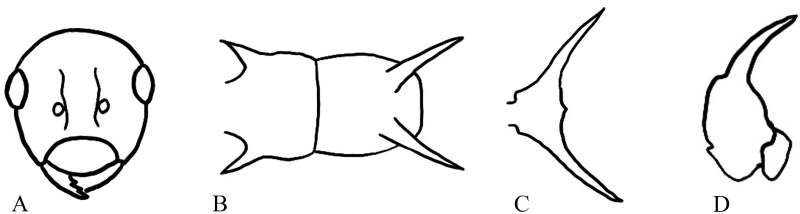
*Polyrhachis jianghuaensis* worker (type). (A) Head in full-face; (B) mesosoma in dorsal view; (C) petiolar in dorsal view; (D) petiolar in lateral view. (Line drawing cited from Wang and Wu 1991).

**Fig. 28.**
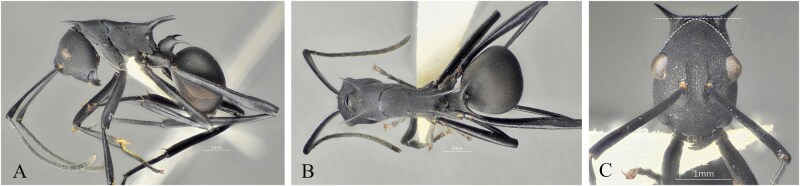
*Polyrhachis bakana* worker (ANTWEB1060616, non-type). (A) Body in lateral view; (B) body in dorsal view; (C) head in full-face. Photographer: Zhenghui Xu.

**Fig. 29.**
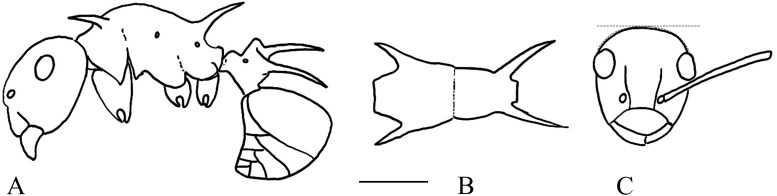
*Polyrhachis zhengi* worker (type). (A) Body in lateral view; (B) mesosoma in dorsal view; (C) head in full-face. (Line drawing cited from Zhou and Huang 2002).

**Fig. 30.**
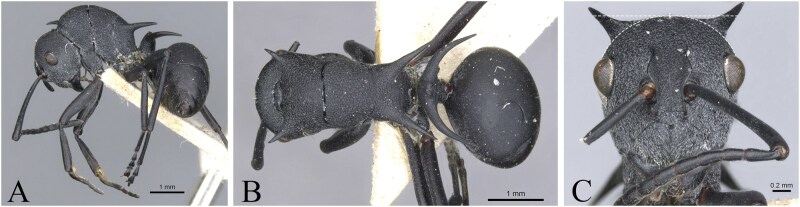
*Polyrhachis thompsoni* worker (CASENT0903383, syntype). (A) Body in lateral view; (B) body in dorsal view; (C) head in full-face. Photographer: Alexandra Westrich.

**Fig. 31.**
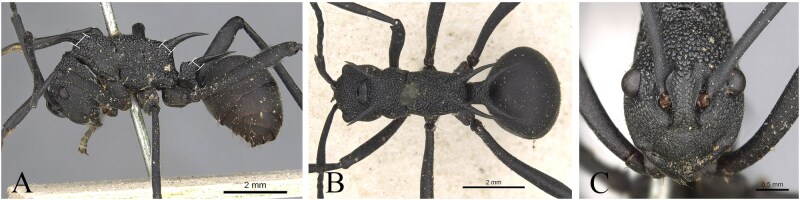
*Polyrhachis armata* worker (CASENT0901854, syntype). (A) Body in lateral view; (B) body in dorsal view; (C) head in full-face. Photographer: Ryan Perry.

**Fig. 32.**
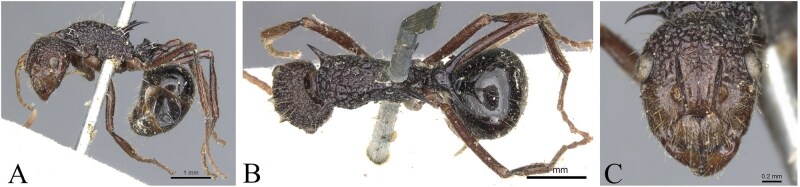
*Polyrhachis rufipes* worker (CASENT0903374, type). (A) Body in lateral view; (B) body in dorsal view; (C) head in full-face. Photographer: Will Ericson.

8. (7) In full-face view, occipital margin strongly convex; anterior clypeal margin with a median emargination (Fig. 26B); propodeal spines robust, their length exceeding that of petiolar spines (Fig. 26A); dorsal margin of petiole concave medially (Fig. 26C)……….*Polyrhachis tianjingshanensis* Qian and Zhou

- In full-face view, occipital margin weakly convex; anterior clypeal margin arcuate (Fig. 27A); propodeal spines slender, their length not exceeding that of petiolar spines (Fig. 27B and D); dorsal margin of petiole convex medially (Fig. 27C)……………………………….*Polyrhachis jianghuaensis* Wang and Wu

9. (2) Propodeal spines uncinate (hook-like) at their apices (Fig. 18)…………………*Polyrhachis mangensis* **sp. nov.** Chen

- Propodeal spines straight and acutely pointed at their apices (Figs 28 and 29)…………………………………………………10

10. (9) Head in full-face view and mesosoma finely and densely puncticulate (Figs 28 and 29)…………………………………11

- Head in full-face view and mesosoma moderately to coarsely punctate (Figs 31 and 32)………………………………………13

11. (10) Head in full-face view, occipital margin strongly convex (more arched; see dashed tangent line) (Fig. 28C); mesosomal dorsum relatively flat in profile (Fig. 28A)…………………………………………………………………….*Polyrhachis bakana* Xu

- Head in full-face view, occipital margin moderately convex (gentler; see dashed tangent line) (Figs 29C and 30C); mesosomal dorsum evenly convex in profile (Figs 29A and 30A)……………………………………………………………12

12. (11) Mesosoma foveolate; petiolar node unarmed between the bases of the petiolar spines; body rufous with gaster black (Fig. 29)………………………………………………………………………*Polyrhachis zhengi* Zhou and Huang

- Mesosoma finely punctate; petiolar node armed with a distinct denticle between the bases of the petiolar spines; body entirely black (Fig. 30)…………………………………………………………………………….*Polyrhachis thompsoni* Bingham

13. (10) Head and mesosoma lacking erect setae (Fig. 31); 3 pairs of spines (pronotal, propodeal, petiolar) robust at base; body black (Fig. 31A)…………………….*P. armata* (Le Guillou)

- Head and mesosoma with medium-density erect setae (Fig. 32); 3 pairs of spines comparatively slender at base; body uniformly rufous (Fig. 32A)…………*Polyrhachis rufipes* Smith

14. (1) Mesosomal dorsum almost straight in profile; body with abundant erect and suberect setae (Figs 33 and 34)………..15

**Fig. 33.**
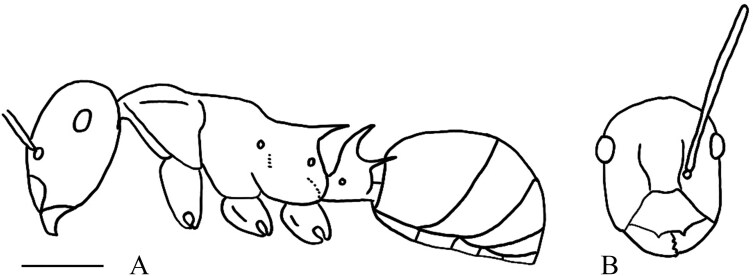
*Polyrhachis euthiacaena* worker (type). (A) Body in lateral view; (B) head in full-face. (Line drawing cited from Zhou and Zheng 1997).

**Fig. 34.**
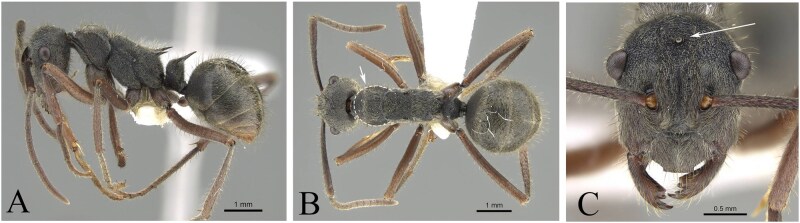
*Polyrhachis lama* worker (CASENT0919909, non-type). (A) Body in lateral view; (B) body in dorsal view; (C) head in full-face. Photographer: Michele Esposito.

**Fig. 35.**
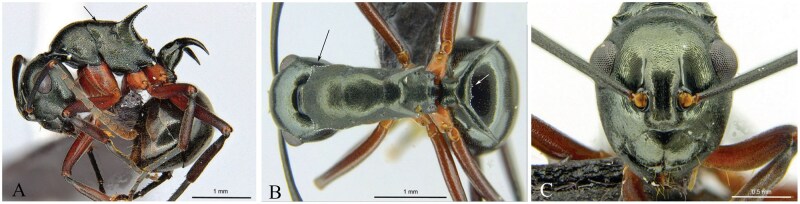
*Polyrhachis hunggeuk* worker (RHL03201, type). (A) Body in lateral view; (B) body in dorsal view; (C) head in full-face. (Images cited from Wong and Guénard 2020).

**Fig. 36.**
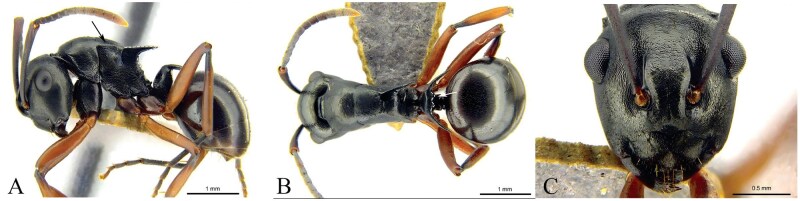
*Polyrhachis confusa* worker (ANTWEB1016626, type). (A) Body in lateral view; (B) body in dorsal view; (C) head in full-face. (Images cited from Wong and Guénard 2020).

-Mesosomal dorsum evenly convex in profile; body lacking conspicuous erect and suberect setae (Figs 35 and 36)…………………………………………………………….16

**Fig. 37.**
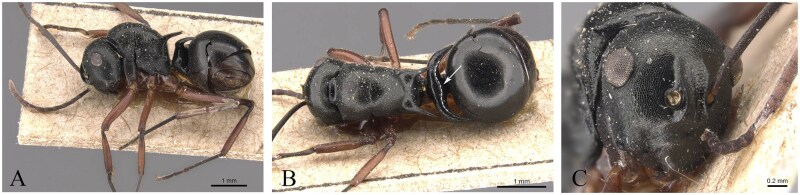
*Polyrhachis lucidula* worker (CASENT0905828, type). (A) Body in lateral view; (B) body in dorsal view; (C) head in full-face. Photographer: Alexandra Westrich.

**Fig. 38.**
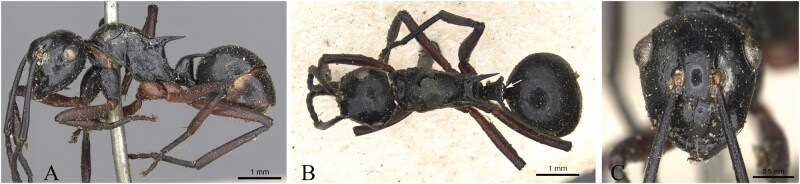
*Polyrhachis laevigata* worker (CASENT0901855, type). (A) Body in lateral view; (B) body in dorsal view; (C) head in full-face. Photographer: Ryan Perry.

**Fig. 39.**
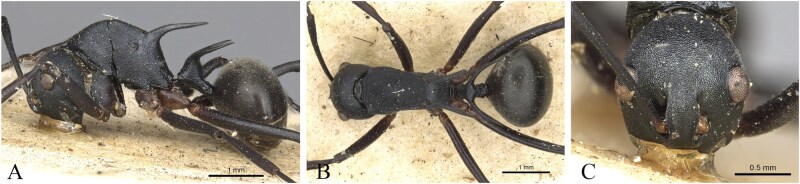
*Polyrhachis hippomanes* worker (CASENT0901873, type). (A) Body in lateral view; (B) body in dorsal view; (C) head in full-face. Photographer: Ryan Perry.

**Fig. 40.**
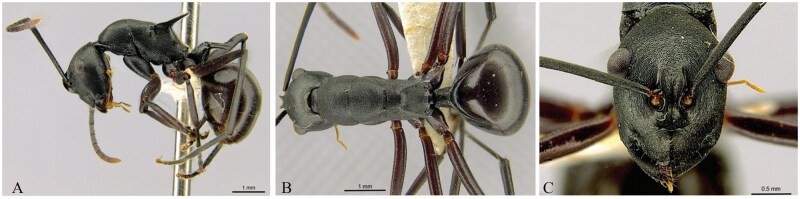
*Polyrhachis peetersi* worker (ANTWEB1015814, type). (A) Body in lateral view; (B) body in dorsal view; (C) head in full-face. (Images cited from Wong and Guénard 2020).

**Fig. 41.**
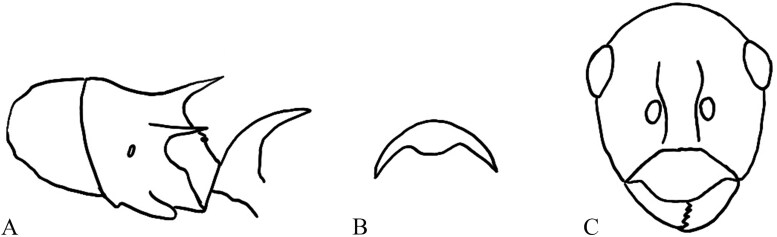
*Polyrhachis rubigastrica* worker (type). (A) Body in lateral view; (B) petiolar in dorsal view; (C) head in full-face. (Line drawing cited from Wang and Wu 1991).

15. (14) Head in full-face view without ocelli; anterior clypeal margin with median emargination (Fig. 33B); petiole triangular in profile (Fig. 33A)……………………………………………………………………….*Polyrhachis euthiacaena* Zhou and Zheng

- Head in full-face view with 3 distinct ocelli; anterior clypeal margin straight (Fig. 34C); petiole rectangular in profile (Fig. 34)……………………………………*Polyrhachis lama* Kohout

16. (14) Dorsal view of petiolar node with 2 distinct denticles between the bases of the dorsal spines (Figs 35 and 36)……….17

- Dorsal view of petiolar node straight or concave between the bases of the dorsal spines, without distinct denticles, or with at most obsolete tubercles (Figs 37 and 38)………………………………………………………………18

17. (16) Mesosomal dorsum immarginate in profile; propodeal spines comparatively slender at base (Fig. 35A)……………………………………….*Polyrhachis hunggeuk* Wong and Guénard

- Mesosomal dorsum distinctly marginate in profile; propodeal spines distinctly broad at base (Fig. 36A)…………………………………………………*Polyrhachis confusa* Wong and Guénard

18. (16) Dorsal view of petiolar node with exceptionally robust apical spines (Fig. 37B)……………….…………….…………….…………….……………………….*Polyrhachis lucidula* Emery

- Dorsal view of petiolar node with comparatively slender apical spines (Figs 38B and 39B)……………………………19

19. (18) In full-face view, area between frontal carinae to clypeus smooth and shining (Fig. 38C)………………………………………………………………….*Polyrhachis laevigata* Smith

- In full-face view, area between frontal carinae to clypeus densely foveolate, dull (Figs 39C and 40C)………………….20

20. (19) Propodeal spines long, slightly curved from base to midlength; petiolar spines long, directed posterodorsally (Fig. 39A and B)………………………………………………………………………………….*Polyrhachis hippomanes* Smith

- Propodeal spines short, straight; petiolar spines short, directed posteriorly (Figs 40 and 41)……………………………………21

21.(20) TL >7 mm; CI <80; humeri armed with larger, sharper denticles (Fig. 40)………………………………………………………………………….*Polyrhachis peetersi* Wong and Guénard

**Fig. 42.**
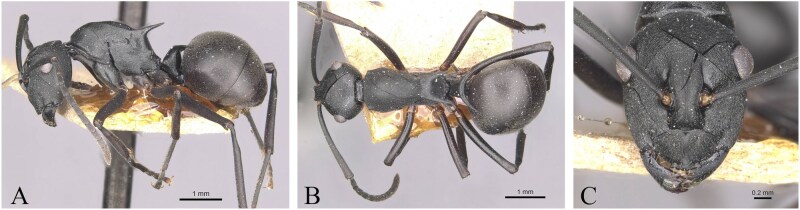
*Polyrhachis moesta* worker (CASENT0910894, type). (A) Body in lateral view; (B) body in dorsal view; (C) head in full-face. Photographer: Will Ericson.

- TL <7 mm; CI >80; humeri with smaller, bluntly angular protrusions (Figs 41 and 42)………………………………………22

22.(21) Frontal carinae narrowly separated, the narrowest point less than 0.2 mm (Fig. 41); scape and gaster testaceous…………………………….*Polyrhachis rubigastrica* Wang and Wu

- Frontal carinae with the narrowest point greater than 0.2 mm; scape and gaster black (Fig. 42)……………………………………………………………………………….*Polyrhachis moesta* Emery

## Discussion

In the present study, we describe 1 new species each from the subgenera *Myrma* and *Myrmhopla: P. aureodorsalis* **sp. nov.** and *P. mangensis* **sp. nov**. Although the genus *Polyrhachis* is exceptionally diverse globally, with approximately 791 described valid species and subspecies, the Chinese fauna currently comprises only 56 species, representing a mere 7% of the world’s total. This disparity underscores that, despite China being a recognized global biodiversity hotspot, a substantial number of species within its borders likely remain undescribed.

The subgenus *Myrma* was comprehensively characterized by Kohout (1989), and its current classification reveals a distinct biogeographic pattern. The Afrotropical fauna is structured into 6 endemic species-groups (Bolton 1973), whereas the Oriental and Australasian fauna is now divided into 9 groups, reflecting more recent revisions (Kohout 2006, 2013, Sorger and Zettel 2009). *Polyrhachis aureodorsalis* **sp. nov.** is confidently assigned to *Myrma* based on its general habitus and pronotal spine structure. Its most diagnostic feature is the unique pilosity on the mesosomal dorsum—a dense covering of golden, appressed, and erect setae. This “golden-back” characteristic immediately distinguishes it from regional congeners and may represent a potential synapomorphy for a species complex within *Myrma.* At present, we have not classified this species into the species group.

The substantial diversity within *Myrmhopla* has long been recognized. When Emery (1925) provided his diagnosis for the subgenus, it already contained approximately 140 species and subspecies, leading him to subdivide it into 6 species-groups in an attempt to manage this diversity. This framework was significantly expanded by Dorow (1995), who recognized 16 species-groups. *Polyrhachis mangensis* **sp. nov.** is provisionally classified within the *Myrmhopla arachne* group based on the morphological species-group definitions of Dorow (1995). This placement is further supported by its ecological association with bamboo leaves, which is shared with other species of this group (Dorow 1995). The discovery represents the first record of this species-group in China. The strongly uncinate propodeal spines of *P. mangensis* **sp. nov.** constitute a remarkable morphological adaptation; we hypothesize that this “hook-like” mechanism may serve as an effective defense against large arthropod predators or small vertebrates, Similar hook-like spines have been documented as effective anti-predator devices in other *Polyrhachis* species (eg *Polyrhachis lamellidens*, *P. bihamata*). However, it should be noted that no molecular phylogenetic study has yet tested the monophyly of the arachne-group; thus, this placement remains a morphological hypothesis requiring future testing with molecular data. Furthermore, *P. mangensis* is currently known only from the mountainous region of Mang City, Yunnan. Whether this indicates a restricted distribution or reflects limited sampling effort remains uncertain, as no comprehensive taxonomic revision of *Polyrhachis* has yet been conducted for China. Recent phylogenomic analyses of the Camponotini by Ward et al. (2025) recovered a well-supported backbone phylogeny for *Polyrhachis*, offering a valuable framework for future studies on the evolution, biogeography, and species-group classification of this genus in China.

*Polyrhachis mangensis* **sp. nov.** is currently known only from the mountainous region of Mang City, Yunnan, where it was collected in bamboo forests. Whether this indicates a restricted distribution or reflects limited sampling effort remains uncertain, and further surveys are needed to clarify its full range. The discovery of *P. aureodorsalis* **sp. nov.** in the mountainous region of western Fujian Province (Youxi County, Sanming City) adds a new member to the local ant fauna. The disjunct distribution patterns between these new species and their closest relatives (eg *P. mangensis* vs. *P. arachne*; *P. aureodorsalis* vs. *P. illaudata*) highlight the potential role of geographic isolation and heterogeneous habitats in southern China in driving speciation within *Polyrhachis*.

This study provides comprehensive, illustrated keys to the Chinese species of *Myrma* and *Myrmhopla*, incorporating the new species and updated diagnostic characters. However, it is acknowledged that some species complexes, particularly those involving widely distributed and morphologically variable taxa, may require future integrative approaches combining molecular data with detailed morphology for robust phylogenetic resolution. A notable limitation of our study is that both new species were collected as foraging workers away from their nests; we did not locate the nests or collect other castes (eg queens, males). Further fieldwork is therefore essential to discover the nests and understand the complete life history of these new species.

## Conclusion

This study brings the number of known Chinese species in the subgenus *Myrma* to 14 and in *Myrmhopla* to 24, including the 2 new species, *P. aureodorsalis* and *P. mangensis*, described herein. The illustrated identification keys provided for these 2 subgenera will serve as a fundamental tool for the accurate identification of Chinese species, facilitating future research and conservation efforts. A notable aspect of this study is that despite repeated collection efforts at the type localities, only foraging workers of the new species were encountered; their nests and other castes remain undiscovered. This gap highlights the elusive nature of these ants and underscores the necessity for further targeted fieldwork to locate their nests, which is crucial for a comprehensive understanding of their colony structure and full life history.

## Nomenclature

This paper and the nomenclatural act it contains have been registered in Zoobank (www.zoobank.org), the official register of the International Commission on Zoological Nomenclature. The LSID (Life Science Identifier) number of the publication is: urn: lsid:zoobank.org:pub:0533B2D3-A677-45A0-8ED8-330C6079C9B2.

## Supplementary Material

ieag070_Supplementary_Data
